# Guinea Pig Model for Lassa Virus Infection of Reproductive Tract and Considerations for Sexual and Vertical Transmission

**DOI:** 10.3201/eid3112.250396

**Published:** 2025-12

**Authors:** Josilene N. Seixas, Joy M. Gary, Stephen R. Welch, Sarah C. Genzer, JoAnn D. Coleman-McCray, Jessica R. Harmon, Christina F. Spiropoulou, Luciana Silva-Flannery, Georgia Ficarra, Elizabeth Lee, Julu Bhatnagar, Jessica R. Spengler, Jana M. Ritter

**Affiliations:** Centers for Disease Control and Prevention, Atlanta, Georgia, USA

**Keywords:** Lassa virus, Lassa fever/pathology, hemorrhagic fevers, viruses, pathology, reproductive tract infections, infectious pregnancy complications, guinea pigs, animal disease models, in situ hybridization, immunohistochemistry

## Abstract

Lassa virus (LASV) causes Lassa fever; mortality rates are higher in pregnant women, and fetal infection and death are possible. Sexual transmission after recovery from Lassa fever has occurred. Using virus strains that are lethal (Josiah) or nonlethal (NJ2015) in guinea pigs, we characterized LASV-associated pathology and reproductive tissue tropism in male and female animals. Uterus, ovary, and epididymis were the earliest and most affected tissues; perivascular lymphocytic inflammation was prominent at lethal timepoints and persisted in survivors after clinical disease. LASV-Josiah RNA was detected in reproductive tissues by 4 days postinfection (dpi). Virus localized by immunohistochemistry and in situ hybridization predominantly within vascular smooth muscle and interstitial mesenchymal cells and was widespread in reproductive tissues in lethal infections (12–25 dpi) but not detected in survivors (41–42 dpi). Using a physiologically relevant model, we describe reproductive tissue targets to further elucidate LASV infection and effects on reproductive health and virus transmission.

Lassa virus (LASV) is a zoonotic arenavirus that causes Lassa fever (LF), a hemorrhagic disease endemic to West Africa, where seasonal outbreaks result in an estimated 1–3 million illnesses and up to 5,000 deaths annually (*1*). After an incubation period of 6–21 days, the disease manifests as fever, weakness, and malaise, followed by musculoskeletal, respiratory, gastrointestinal, or other signs. Long-term ocular (*2*,*3*) and auditory (*4*–*6*) complications can occur. In severe cases, hemorrhagic fever with shock causes death within 14 days (*7*). Mortality rates in pregnant women are disproportionately high, especially in the third trimester, in which reported mortality rates were >2 times those observed in nonpregnant women (*8*). Fetal loss rates are 75%–100% (*9*,*10*), and vertical transmission has been reported (*9*–*12*). 

Most LF is caused by rodent-to-human transmission through direct contact with infected animals or their excreta (*13*–*17*). Horizontal human transmission, mostly nosocomial, has also been reported through contact with infected blood, urine, or other body fluids (*18*). Sexual transmission of LASV has been postulated (*19*), and persistence of LASV in seminal fluid for >3 months has been documented; however, studies describing the dynamics and infectivity of LASV in semen or other reproductive tract secretions are sparse (*12*,*20*).

Autopsies are rarely performed on LF patients, and reproductive tissues are not routinely collected; thus, descriptions of fatal LF pathology and tissue virus distribution are limited overall, and particularly for reproductive tissues (*21*). In 1 human study, pathology associated with LASV infection was described for 6 complete autopsies, 15 cases with postmortem biopsies of tissues other than liver, and 7 fetuses from infected women (*22*). The authors mentioned that careful examination of ovary, uterus, placenta, and breast demonstrated no specific pathologic alterations (*22*). Another study of postmortem tissue samples from 12 confirmed LF cases reported LASV antigens and viral particles in multiple reproductive organ and cell types, including breast ductal epithelial cells, ovarian thecal and stromal cells, and placental trophoblasts (*21*). To address gaps in knowledge of reproductive tract effects of LASV infection and to learn more about potential for sexual transmission, we investigated LASV pathology and tissue tropism in the female and male reproductive tracts of experimentally infected strain 13/N guinea pigs, a well-characterized model of LASV disease (*2*,*3*,*23*,*24*) and an applicable model of human pregnancy and fetal growth (*23*).

## Materials And Methods

### Biosafety and Ethics

Work with infectious virus or infected animals was conducted in a Biosafety Level 4 laboratory at the Centers for Disease Control and Prevention (CDC; Atlanta, GA, USA). Recombinant virus work was approved by the CDC Institutional Biosafety Committee. Animal experiments were approved by the CDC Institutional Animal Care and Use Committee (approval nos. 2833, 3073) and performed in an AAALAC-accredited facility.

### Virus and Virus Detection

Recombinant LASV-Josiah is based on the sequence of an isolate obtained in 1976 from the serum of a 40-year-old man hospitalized at Songo Hospital in Sebgwena, Sierra Leone (*24*,*25*). We rescued LASV-Josiah in BSR-T7/5 cells and passaged 2 times in Vero-E6 cells (GenBank accession nos. HQ688673.l, HQ688675.1). Recombinant LASV 812673-LBR-USA-2015 (LASV-NJ2015) is based on the sequence of an isolate obtained in 2015 from a 55-year-old man who died of LF in New Jersey after returning from Liberia. We rescued LASV-NJ2015 in BSR-T7/5 cells and passaged 2 times in Vero-E6 cells (*26*) (GenBank accession nos. MG812650 and MG812651). We sequence-verified viral stocks and confirmed them to be mycoplasma-free. We determined titers (as focus-forming units [FFU] or 50% tissue culture infectious dose per milliliter) on Vero-E6 cells by immunofluorescence assays using an in-house anti-LASV monoclonal antibody mix (SPR628) targeting nucleoprotein (NP) and glycoprotein (GP) 2 and calculated them using the method of Reed and Muench (*27*). Further information on virus detection in tissues by quantitative reverse transcription PCR (qRT-PCR), immunohistochemistry (IHC), and in situ hybridization (ISH) are provided (Appendix). 

### Guinea Pigs

**Figure 1:**
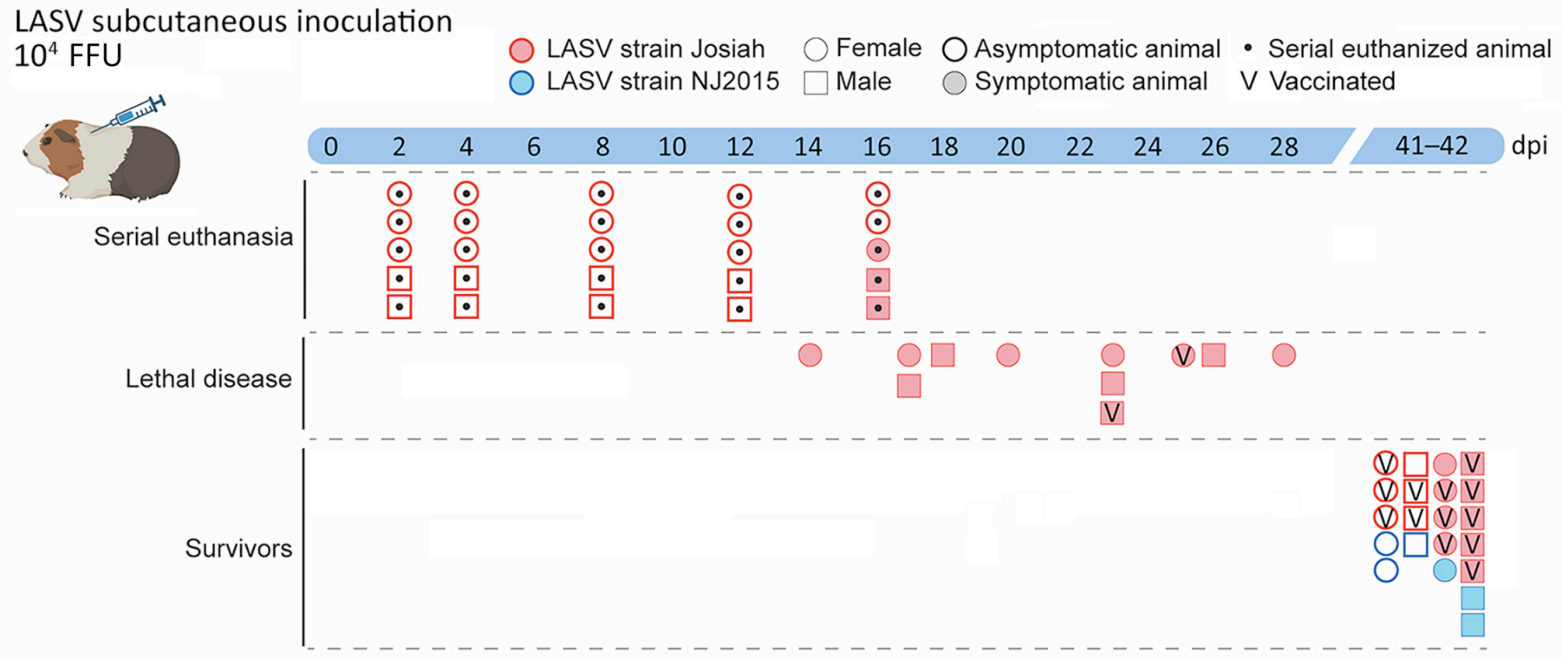
Study design to evaluate reproductive tissue pathology and tissue tropism in strain 13/N guinea pig model of Lassa virus infection. Tissues collected from a total of 57 adult strain 13/N guinea pigs inoculated subcutaneously with a target dose of 10^4^ FFU of LASV (equivalent to ≈2 × 10^4^ 50% tissue culture infectious dose;, representing 3 independent studies, were examined by PCR, hematoxylin and eosin, immunohistochemistry, or in situ hybridization. Animals were separated into 3 study groups on the basis of time of sampling and clinical outcome: those serially euthanized at predetermined study endpoints (2, 4, 8, 12, or 16 dpi [n = 5 at each timepoint]) to investigate early infection with highly pathogenic LASV-Josiah strain; terminal animals that reached endpoint criteria because of disease after LASV-Josiah infection (unvaccinated [n = 9] or vaccinated with 𝛾-irradiated viral replicon particle [VRP] [n = 2]); and survivors euthanized at 41–42 dpi. Survivors were 15 animals infected with LASV-Josiah (vaccinated preexposure with VRP [n = 5] or gamma-irradiated VRP [n = 3], vaccinated postexposure with VRP [n = 5], or unvaccinated [n = 2] and 6 animals infected with strain NJ2015 [all unvaccinated (n = 6)]). Shaded symbols indicate animals that exhibited overt signs (defined as weight loss >10%, temperature >39.5°C for >2 consecutive days, or presence of clinical signs [i.e., clinical score >1 on >1 days). dpi, days postinfection; FFU, focus-forming units; LASV, Lassa virus; V, vaccinated.

We examined tissues from 57 strain 13/N guinea pigs (26 male and 31 female, age range 165–1,433 days at challenge) previously reported in pathogenesis (*26*,*28*–*30*) or vaccine (*31*) studies. We inoculated all animals subcutaneously between the scapulae with 10^4^ FFU (equivalent to ≈2 × 10^4^ 50% tissue culture infectious dose/mL) of recombinant LASV-Josiah (n = 51 [23 male, 28 female]) or LASV-NJ2015 (n = 6 [3 male, 3 female]). Fifteen of 51 LASV-Josiah–inoculated animals were included in a viral replicon particle (VRP) vaccine study (*31*), in which animals were vaccinated subcutaneously with 10^7^ FFU of VRP under these conditions: VRP followed by challenge 28 days later (n = 5); replication incompetent VRP (treated with 5 × 10^6^ rads of gamma-irradiation) followed by challenge 28 days later (n = 5); and VRP 1 day after challenge (n = 5) (Figure 1). All remaining animals (n = 42) inoculated with either LASV-NJ2015 (n = 6) or LASV-Josiah (n = 36) were unvaccinated. Animals were housed individually in a climate-controlled laboratory (68–79°F, 30%–70% humidity) on a 12-hour light/dark cycle in individually ventilated cages (Thoren Caging Systems Inc., https://thoren.com) with deep, soft bedding (soft pellets, Carefresh [Healthy Pet, https://www.healthy-pet.com], and Enviro-Dri [Shepherd Specialty Papers, https://www.ssponline.com]). Animals received daily fresh vegetable enrichment and Timothy hay, and Certified Guinea Pig Diet 5026 (LabDiet, https://www.labdiet.com) and water were provided ad libitum. For individual identification and body temperature monitoring, we implanted BMDS IPTT-300 microchip transponders (Avidity Science, https://www.avidityscience.com) subcutaneously in the interscapular region. We monitored animals daily, as previously reported (*26*,*30*,*31*) and humanely euthanized them with isoflurane vapors followed by intracardiac sodium pentobarbital injection upon meeting euthanasia criteria or at study completion. 

## Results

### Widespread Reproductive Tract Infection during Lethal LASV Disease in Guinea Pigs

**Figure 2:**
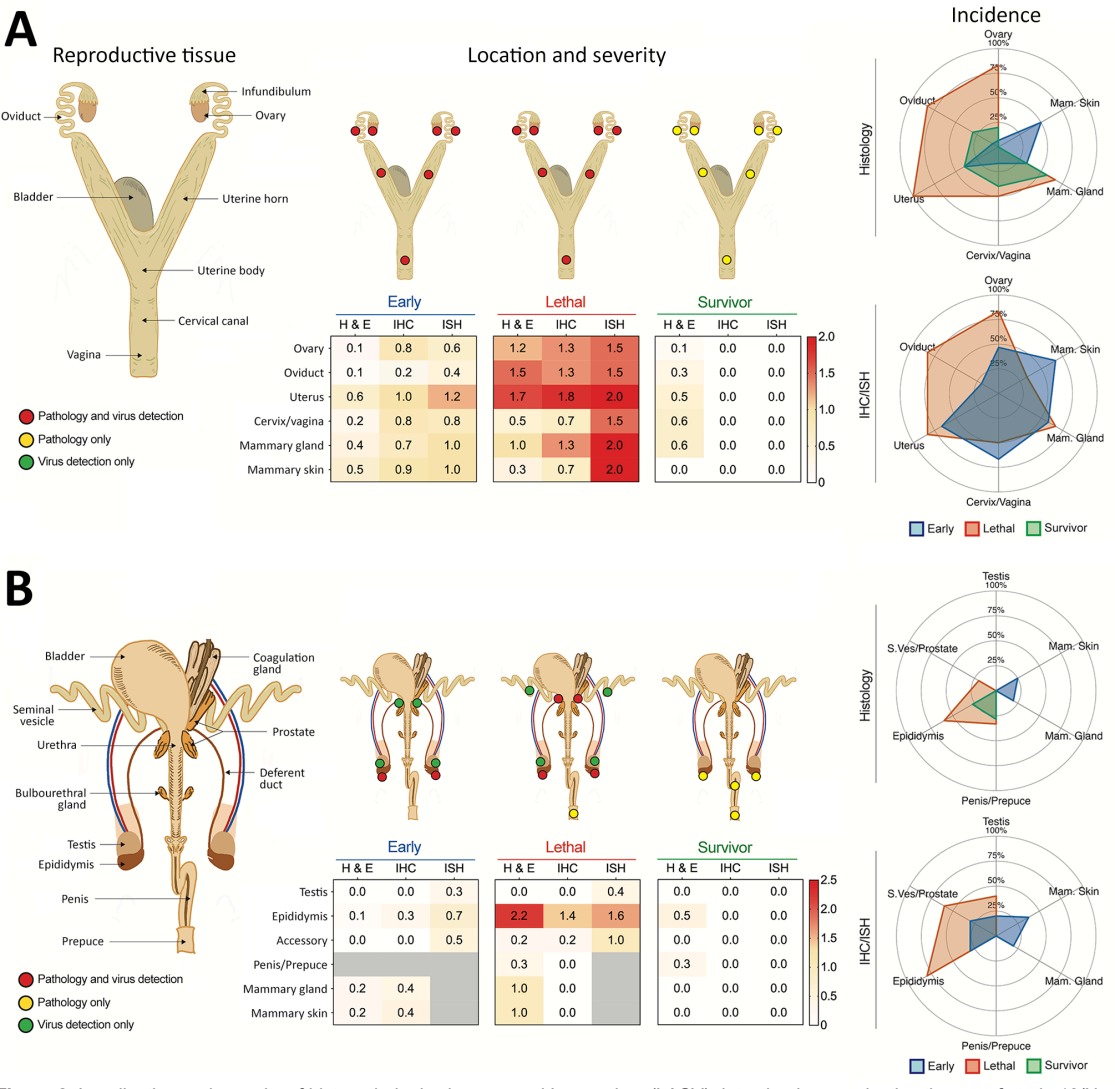
Localization and severity of histopathologic changes and Lassa virus (LASV) detection in reproductive tissues of strain 13/N guinea pigs experimentally infected with LASV strain Josiah or NJ2015 in study of guinea pig model for LASV infection of reproductive tract and considerations for sexual and vertical transmission. Female (A) and male (B) guinea pig reproductive tract anatomy, histopathology, and virus detection (antigen by IHC or viral RNA by ISH) after subcutaneous infection with LASV strain Josiah or NJ-2015 (target dose, 10^4^ focus-forming units). Anatomic localization, severity, and incidence are depicted and delineated by stage of infection: early (<16 dpi), lethal (meeting endpoint criteria at 14–28 dpi), and survivor (41 or 42 dpi). Histopathologic changes (H&E) and viral detection by (IHC or ISH) were scored semiquantitatively for each tissue as absent (0), minimal (1), mild (2), moderate (3), or severe (4). Not all analyses were performed for all animals (Appendix Tables 1–3, https://wwwnc.cdc.gov/EID/article/31/12/25-0396-App1.pdf). H&E, IHC, and ISH scores represent mean severity values for each tissue across all animals tested. An asterisk (*) indicates that only 1 animal was evaluated in the group; gray boxes indicate tissues that were not available or not evaluated. Relative incidence of histopathologic change or virus detection in early, lethal, and survivor cohorts with sufficient group sizes (n >3) is shown in radar plots; only a single male animal in the lethal cohort was excluded, as it was the sole animal evaluated and test results were positive in both mammary gland and skin. H&E, hematoxylin and eosin; IHC, immunohistochemistry; ISH, in situ hybridization.

**Figure 3:**
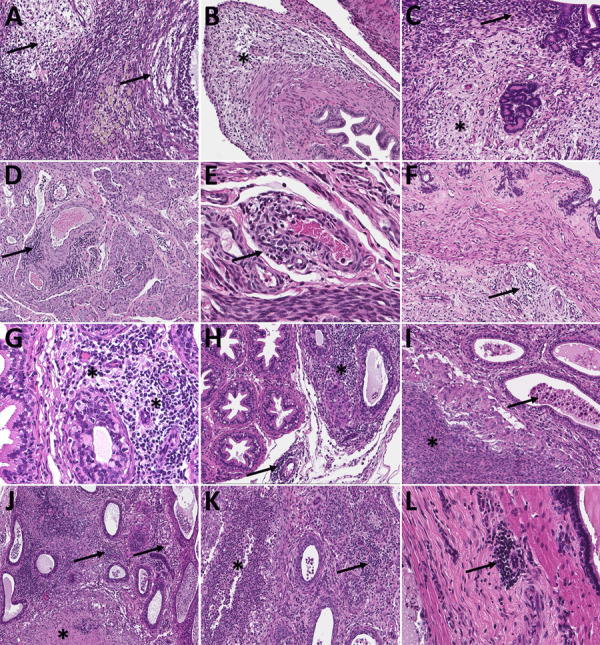
Histopathologic findings in reproductive tissues of strain 13/N guinea pigs with lethal disease after Lassa virus (LASV) strain Josiah infection in study of guinea pig model for LASV infection of reproductive tract and considerations for sexual and vertical transmission. Panels A–F depict samples from female guinea pigs; panels G–L depict samples from male guinea pigs. A) Ovary (25 days postinfection [dpi]) with moderate perivascular and interstitial inflammation (arrows). B) Oviduct (20 dpi) with focal interstitial inflammation and edema (asterisk) within the serosa. C) Uterus (23 dpi) with mild endometrial inflammation and edema (asterisk) and hemosiderin-laden macrophages (arrow). D) Uterus (25 dpi) with multifocal mononuclear inflammatory cells (arrow) forming partial cuffs around vessels in the myometrium. E) Uterine vessel (23 dpi) with mural infiltration by lymphocytes and plasma cells (arrow). F) Cervix (23 dpi) with multifocal, mild, perivascular inflammation (arrow). G) Epididymis (26 dpi) with moderate perivascular inflammation (asterisks). H) Epididymis (23 dpi) with multifocal interstitial inflammation around tubules (asterisk) and a vessel (arrow). I) Epididymis (26 dpi) with marked epididymitis with tubular dilation by inflammatory cells (arrow) and rupture, associated with sperm granuloma formation (asterisk). J) Epididymis (23 dpi) with marked epididymitis, with interstitial inflammation (arrows), and intratubular heterophils associated with rupture and granuloma formation (asterisk). K) Epididymis (26 dpi) with marked interstitial inflammation (arrow) and tubular epithelial necrosis with heterophils (asterisk). L) Prostate (23 dpi) with mild focal lymphocytic, perivascular inflammation (arrow). Hemotoxylin and eosin stain. Original magnifications ×20 (panels A, E, G, I, K), ×15 (panels B, H), and ×10 (panels C, D, F, J, L).

To investigate reproductive tract infection histologically in lethal disease, we collected tissues from strain 13 guinea pigs at terminal endpoint after subcutaneous LASV-Josiah inoculation, which caused severe disease warranting euthanasia 14–28 days postinfection (dpi) (median 23 days) in 11 animals (6 female, 5 male). Inflammatory changes were identified in >1 reproductive tissue from all (6/6) female and 3/5 male animals, including ovary, oviduct, uterus, cervix/vagina, epididymis, seminal vesicle, and penis/prepuce; inflammation was not seen in testes (Table; Figures 2, 3; Appendix Table 1). Those tissues had mild to moderate, multifocal inflammation, which formed perivascular cuffs with occasional vascular mural infiltration. We noted more inflammation at 20–26 dpi than at 14–17 dpi; less inflammation was present at the latest lethal timepoint (28 dpi; 1 female). Infiltrates were predominately lymphocytic and presence of plasma cells and histiocytes was variable; heterophils were rare (Table). 

**Table:** Pathologic findings and virus localization by immunohistochemistry or in situ hybridization in reproductive and mammary tissues of LASV-Josiah or LASV-NJ2015 strain–infected guinea pigs at different timepoints in study of guinea pig model for LASV infection of reproductive tract and considerations for sexual and vertical transmission*

Sex	Tissue	Group†	Hematoxylin and eosin			Immunohistochemistry or in situ hybridization	
			n/N (%)‡	Lesion/change		n/N (%)‡	Cellular location
F	Ovary	Early	1/15 (7)	PILPI, PE		7/15 (47)	TIC, GC
		Lethal	5/6 (83)	PILPI, PE		5/6 (83)	TIC, GC, IMC, VSMC, ENDC
		Survivor	2/10 (20)	PILPI		0/10 (0)	ND
	Oviduct	Early	1/15 (7)	PILPI		3/15 (20)	IMC
		Lethal	5/6 (83)	PILPI, PE, congestion		5/6 (83)	PV/IMC, VSMC, OSMC, intravascular inflammatory cells
		Survivor	3/10 (30)	PILPI		0/10 (0)	ND
	Uterus	Early	6/15 (40)	PILPI, PIE, congestion		10/15 (67)	Endometrial stromal, epithelial cells, IFMC, ENDC
		Lethal	6/6 (100)	PILPI, PIE, congestion, vasculitis		6/6 (100)	PV/IMC, IFMC, ENDC, VSMC, OSMC, endometrial stromal
		Survivor	4/10 (40)	PILPI, PIE, congestion		0/10 (0)	ND
	Cervix/ vagina	Early	2/12 (17)	PILPI, PIE		8/12 (67)	IMC, VSMC, epithelial cells, intraluminal debris, FMC
		Lethal	3/6 (50)	PILPI, PIE		3/6 (50)	IMC, VSMC, epithelial cells, ENDC, intraluminal debris, IFMC
		Survivor	4/10 (4)	PILPI, PIE		0/10 (0)	ND
M	Testis	Early	0/10 (0)	None		2/10 (20)	IMC, ENDC, intravascular
		Lethal	0/5 (0)	None		2/5 (40)	IMC, seminiferous tubule
		Survivor	0/11 (0)	None		0/11 (0)	ND
	Epididymis	Early	1/10 (10)	PILPI, epithelial necrosis, intratubular debris		3/10 (30)	IMC, peritubular, tubular epithelium, intraluminal cells (including spermatozoa)
		Lethal	3/5 (60)	PILPI, epithelial necrosis, intratubular heterophils		4/5 (80)	IMC, VSMC, ENDC, tubular epithelium, intraluminal cells/debris (including spermatozoa)
		Survivor	3/11 (27)	PILPI, PIE, intratubular heterophils, MXI, loss of tubules		0/11 (0)	ND
	Seminal vesicle/prostate	Early	0/10 (0)	None		3/10 (30)	PV/IMC
		Lethal	1/5 (20)	PILPI, PE		3/5 (60)	IMC, intraluminal debris, VSMC
		Survivor	0/11 (0)	None		0/11 (0)	ND
	Penis/prepuce	Early	NS	NS		NS	NS
		Lethal	1/3 (33)	MXI (preputial gland duct)		0/3 (0)	ND
		Survivor	2/7 (29)	PLPI		0/7 (0)	ND
Both	Mammary tissue	Early	8/20 (40)	PILPI (glands and skin), histiocytic infiltrates, PIE (glands), epidermal necrosis		11/20 (55)	IMC, dermis, follicular wall, epidermis, IFMC
		Lethal	3/4 (75)	PILPI, PIE (glands and skin)		2/4 (50)	IMC, ENDC, glandular epithelial cells, IFMC, intraluminal debris, epidermis, follicular wall
		Survivor	4/13 (31)	PILPI (glands and skin), PIE, congestion, MXI (glands)		0/13 (0)	ND

*dpi, days postinfection; ENDC, endothelial cells; GC, granulosa cells; IFMC, inflammatory cells; IMC, interstitial mesenchymal cells; LASV, Lassa virus; MXI, mixed inflammatory infiltrate; ND, not detected; NS, not sampled; PE, perivascular edema; PIE, perivascular and interstitial edema; PILPI, perivascular and interstitial lymphoplasmacytic inflammation; PLPI, perivascular lymphoplasmacytic inflammation; PV, perivascular; OSMC, other (nonvascular) smooth muscle cells; TIC, Theca-interstitial cells; VSMC, vascular smooth muscle cells. †Early refers to animals serially euthanized 2–16 dpi after infection with LASV-Josiah-strain. Lethal refers to animals lethally infected with LASV-Josiah-strain and euthanized because of severity of clinical disease 14–28 dpi. Survivor refers to animals that survived LASV-Josiah-strain or LASV-NJ2015 strain infection and were euthanized 41–42 dpi. ‡n indicates number of animals with pathologic changes or staining in the tissue; N represents total number of animals evaluated in the group; percentage indicates percentage of animals evaluated that had the histopathologic finding present or with positive staining by immunohistochemistry or in situ hybridization in that tissue; not all tissues were evaluated or tested by immunohistochemistry or in situ hybridization for all animals (Appendix Tables 1–3, https://wwwnc.cdc.gov/EID/article/31/12/25-0396-App1.pdf).

**Figure 4:**
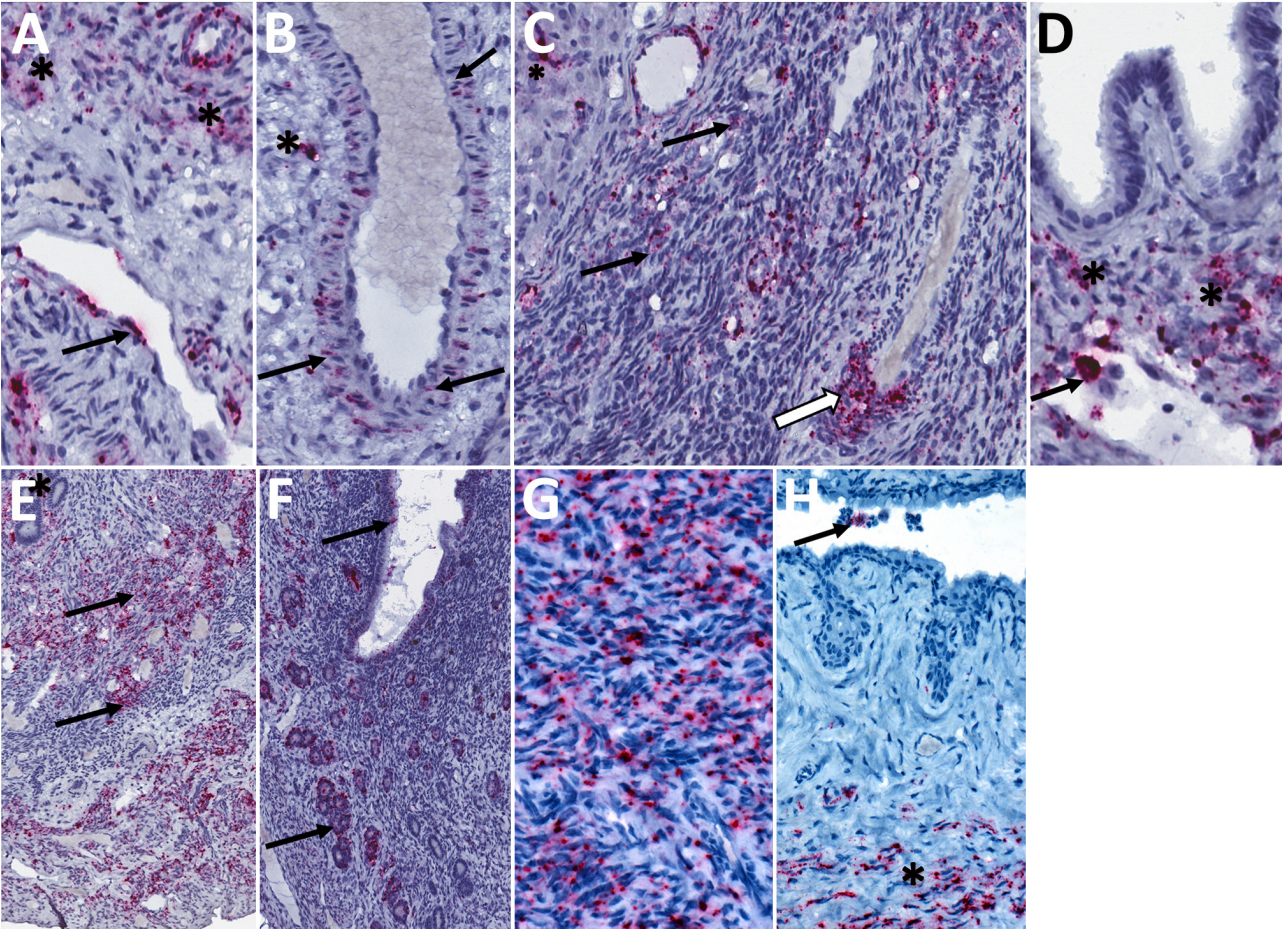
Detection of Lassa virus (LASV) antigens and RNA in reproductive tissues by immunohistochemistry (IHC) and in situ hybridization (ISH) in strain 13/N female guinea pigs lethally infected with LASV strain Josiah in study of guinea pig model for LASV infection of reproductive tract and considerations for sexual and vertical transmission. IHC and ISH chromogens are red. A) Uterus (25 dpi), ISH. Staining in interstitial mesenchymal cells (asterisks) and rare endothelial cells (arrow). B) Uterus (23 dpi), ISH. Staining in vascular smooth muscle cells (arrows) and perivascular interstitial mesenchymal cells (asterisk). C) Ovary (23 dpi), ISH. Staining within a corpus luteum (asterisk), in theca-interstitial cells (arrows), and in inflammatory cells within and around a vascular wall (white arrow). D) Oviduct (23 dpi), ISH. Staining in mesenchymal interstitial cells (asterisk) and intravascular inflammatory cells (arrow). E) Uterus (23 dpi), ISH. Extensive staining in the endometrial stroma (asterisk) and myometrial smooth muscle cells (arrows). F) Uterus (23 dpi), ISH. Extensive staining in the endometrial glandular epithelial cells (arrows). G) Endocervix (23 dpi), IHC. Extensive staining in mesenchymal cells. H) Vagina (23 dpi), IHC. Staining in submucosal mesenchymal cells (asterisk) and intraluminal cellular debris (arrow). Original magnifications ×40 (panels A, B, D, G, H), ×30 (panel C), ×20 (panels E, F).

Among 6 female animals with lethal disease, we detected viral antigen, RNA, or both in ovary, oviduct, and uterus of all 5 animals that were euthanized on days 14–25 and in the cervix/vagina in 3/5 animals tested (Figure 2, panel A; Appendix Table 1). Staining was most frequent in the vascular smooth muscle and perivascular mesenchymal cells including fibroblasts, as well as in the uterine muscle and endometrial stroma (Table, Figure 4). We saw similar distribution and intensity of staining in all positive animals; 1 female animal euthanized at 28 dpi had only very mild inflammation and no antigen was detected in reproductive tissues, although in situ hybridization (ISH) was positive in uterus. 

**Figure 5:**
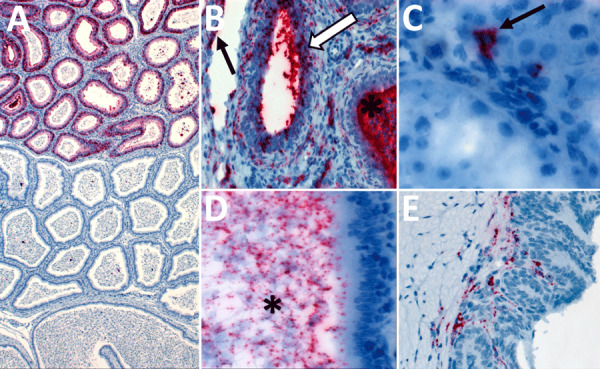
Detection of Lassa virus (LASV) antigens and RNA in reproductive tissues by immunohistochemistry (IHC) and in situ hybridization (ISH) in strain 13/N male guinea pigs lethally infected with LASV strain Josiah in study of guinea pig model for LASV infection of reproductive tract and considerations for sexual and vertical transmission. A) Epididymis (18 dpi), ISH. Regionally extensive staining in epididymal epithelial cells. B) Epididymis (26 dpi), ISH. Staining in interstitial mesenchymal cells, endothelium (arrow), tubular epithelium (white arrow), and intratubular inflammatory cells (asterisk). C) Testis (18 dpi), ISH. Focal intracellular staining in the interstitium (arrow). D) Epididymis (23 dpi), ISH. Intraluminal staining including spermatozoa (asterisk). E) Seminal vesicle (17 dpi), ISH. Focal staining in subepithelial stromal cells. Original magnifications ×5 (panel A), ×40 (panels B, D), ×63 (panel C), ×20 (panel E).

Among male animals with lethal disease, viral antigen was detected in the epididymis of 4/5 animals euthanized at 17–26 dpi (Figure 2, panel B; Appendix Table 1). Epididymal staining was focally extensive and within interstitial stromal and inflammatory cells, as well as tubular epithelium, endothelium, vascular smooth muscle cells, and intratubular cells including spermatozoa (Table; Figure 5). We detected no antigen in any testis from lethally infected male animals, and only 2/5 had rare granular ISH staining in very few seminiferous epithelial or interstitial cells. In addition, staining by immunohistochemistry (IHC) or ISH occurred very rarely in 3 accessory sex glands (2 seminal vesicles, 1 prostate) of lethally infected male animals at 17–23 dpi. 

We quantified viral RNA (vRNA) using qRT-PCR on ovary and testis samples from a subset (3 female, 3 male) of lethally infected animals. Testing revealed vRNA in all: up to 2.2 × 10^7^ copies/µL in ovaries and up to 4.3 × 10^3^ copies/µL in testes.

**Figure 6:**
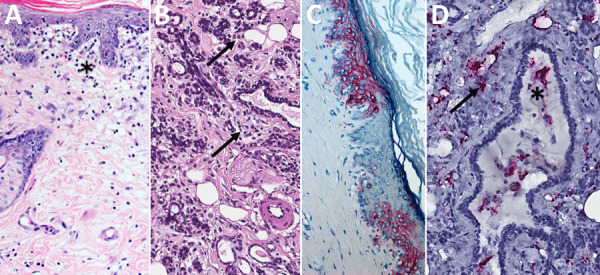
Histopathologic findings and detection of Lassa virus (LASV) antigens and RNA in mammary tissues by immunohistochemistry (IHC) and in situ hybridization (ISH) in strain 13/N guinea pigs lethally infected with LASV strain Josiah in study of guinea pig model for LASV infection of reproductive tract and considerations for sexual and vertical transmission. A) Mammary skin (23 dpi), hematoxylin and eosin stain. Mild interstitial lymphoplasmacytic infiltrates and edema (asterisk) in the superficial dermis. B) Mammary gland (25 dpi), hematoxylin and eosin stain. Mild interstitial lymphoplasmacytic infiltrates (arrows) in the periglandular stroma. C) Mammary skin (23 dpi), IHC. Staining in keratinocytes of the epidermis. D) Mammary gland (23 dpi), ISH. Staining around vessels and within the stroma between glands (arrow) and within cells in a mammary duct lumen (asterisk). Original magnifications ×10 (panels A–C), ×20 (panel D).

We evaluated mammary tissues in 3/6 female and 1/5 male animals with lethal disease. Two female and 1 male animals had mild glandular interstitial or dermal lymphocytic infiltrates and edema (Table; Figure 6). We detected antigen or RNA in mammary tissue from the 2 female animals, localized to rare endothelial cells and perivascular cells including stromal and inflammatory cells, glandular epithelium and intraluminal cells, and epidermal and follicular epithelium (Table; Figure 6).

### Detection of LASV-Josiah in Reproductive Tissues before Onset of Clinical Signs

After LASV-Josiah challenge, we collected tissues for histologic and virologic (qRT-PCR) investigation in animals serially euthanized at 2, 4, 8, 12, or 16 dpi to characterize early reproductive tract infection. In female animals, we detected vRNA by qRT-PCR in mammary tissue as early as 4 dpi and in ovary (and in urogenital swab specimens, as previously reported [*30*]) from 8–16 dpi. In male animals, we detected vRNA by qRT-PCR in testis, epididymis, mammary tissue, and genital swabs from 8–16 dpi. At 8 dpi, vRNA was detected by qRT-PCR in the gonads and associated reproductive tissues of all serially euthanized animals (ovaries, ≤7.3 × 105 copies/μL; uteri, ≤5.5 × 104 copies/μL; testes, ≤1.0 × 105 copies/μL; and epididymides, ≤3.4 × 104 copies/μL).

**Figure 7:**
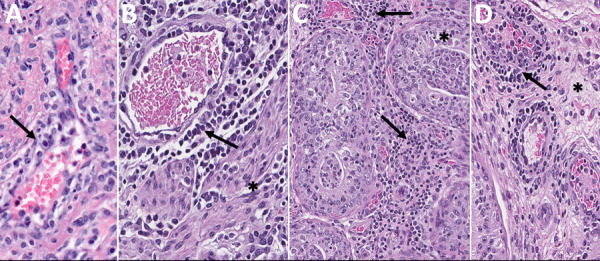
Histopathologic findings in reproductive tissues from strain 13/N guinea pigs serially euthanized 4–16 days postinfection (dpi) after Lassa virus strain Josiah infection in study of guinea pig model for infection of reproductive tract and considerations for sexual and vertical transmission. A) Perivascular mononuclear infiltrates (arrow) in oviduct (12 dpi). B) Perivascular (arrow) and interstitial (asterisk) lymphoplasmacytic infiltrates within the myometrium in uterus (16 dpi). C) Interstitial and perivascular lymphoplasmacytic infiltrates (arrows), with rare single cell necrosis of the tubular epithelial cells (asterisk) in epididymis (16 dpi). D) Mild perivascular and vascular inflammation (arrow) and interstitial edema (asterisk) in epididymis (16 dpi). Hematoxylin and eosin stain. Original magnifications ×40 (panels A, B, D), ×20 (panel C).

Histopathologic changes were absent in reproductive tissues of serially euthanized animals at earlier timepoints (female at 2–8 dpi, male at 2–12 dpi); we detected only subtle changes, similar but overall milder than seen in lethal disease, in 6/6 uteri and rarely in other tissues (1 ovary, 1 oviduct, 2 cervix/vagina) of female animals euthanized at later timepoints (12–16 dpi). One of 2 male animals had lymphoplasmacytic inflammation with rare epithelial necrosis and intratubular debris in the epididymis at 16 dpi (Table; Figures 2, 7; Appendix Table 2). We saw no other notable histopathologic lesions in the male reproductive tracts in serially euthanized animals.

**Figure 8:**
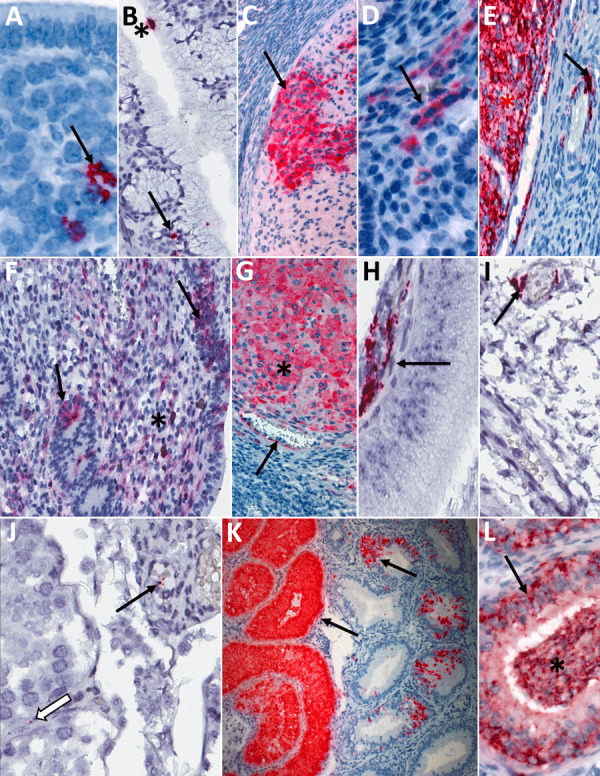
Detection of viral antigens and RNA by immunohistochemistry (IHC) and in situ hybridization (ISH) in reproductive tissues from strain 13/N guinea pigs serially euthanized 4–16 dpi after LASV strain Josiah infection in study of guinea pig model for infection of reproductive tract and considerations for sexual and vertical transmission. IHC and ISH chromogens are red. Panels A–G depict samples from female guinea pigs; panels H–L depict samples from male guinea pigs. A) Uterus (4 dpi), ISH. Rare staining in the endometrial stroma (arrow), without inflammation. B) Endocervix (4 dpi), ISH. Staining of the apical surface of an endocervical epithelial cell (asterisk) and beneath the basilar epithelium (arrow), without inflammation. C) Ovary (8 dpi), IHC. Staining in granulosa lutein cells (arrow) of a corpus luteum without inflammation. D) Uterus (8dpi), IHC. Staining in endometrial stromal cells (arrow) without inflammation. E) Ovary (12 dpi), ISH. Staining in theca interstitial cells (asterisk) and endothelium (arrow). F) Uterus (12 dpi), IHC. Endometrial stromal lymphoplasmacytic infiltrates and staining in endometrial stromal (asterisk) and epithelial (arrows) cells. G) Ovary (16 dpi), IHC. Staining in granulosa cells (asterisk) and endothelium (arrow). H) Epididymis (8 dpi), IHC. Staining in peritubular interstitial mesenchymal cells (arrow). I) Seminal vesicle (12 dpi), ISH. Staining in the wall of a small interstitial vessel (arrow). J) Testis (12 dpi), ISH. Rare granular staining of viral RNA in the interstitium (white arrow) and in a vascular lumen (arrow). K, L) Epididymis (16 dpi), ISH. Extensive staining in tubular epithelium (arrows) and within tubular lumens, including spermatozoa (asterisk). Original magnifications ×40 (panels A, C, D, F, G, H, J, L), ×63 (panels B, I), ×10 (panel E), ×4 (panel K).

In serially euthanized female animals, 10/15 had staining by IHC or ISH in >1 reproductive tissue (Table; Figures 2, 8; Appendix Table 2). We observed staining in 7/9 ovaries at 8–16 dpi, the most widespread at 12 dpi. At 8 dpi, focal staining was in a corpus luteum, mainly in granulosa lutein cells, but also in capillary endothelial and few thecal cells. At later time points (at and after 12 dpi), staining was in theca-interstitial cells and granulosa cells. We noted scattered staining in interstitial cells in 3/6 oviducts at 12 and 16 dpi. During 4–16 dpi, 10/12 uteri demonstrated staining. At 4 and 8 dpi, staining was multifocal and rare within endometrial stromal cells. At 12 and 16 dpi, staining in the uterus was similarly distributed in endometrial stromal and epithelial cells but increased overall at 12 dpi and decreased again at 16 dpi. At those later timepoints, staining within inflammatory or endothelial cells in areas with perivascular inflammation was rare. Of available cervix/vagina tissues, 8/11 from 4–16 dpi showed multifocal staining throughout the superficial subepithelial stroma, within endothelial cells and vascular smooth muscle cells, in the myometrium, and, less frequently (2 animals at 12 and 16 dpi), in the cervical epithelium, but not in the vaginal epithelium (Appendix Table 2).

In serially euthanized male animals, 2/10 testes (at 12 dpi) and 3/10 accessory sex glands (at 12 and 16 dpi) showed rare viral detection by ISH but not by IHC. Staining was in interstitial and intravascular cells in those tissues and was unaccompanied by pathologic changes. Epididymides showed multifocal LASV staining by ISH in 3/6 animals tested at 8–16 dpi; only 1 of those 6 also showed staining by IHC at 16 dpi. Staining was in epididymal interstitial cells at 8 dpi and in tubular epithelial and intraluminal cells (including spermatozoa) at 16 dpi. We saw no staining in any tested male tissue before 8 dpi (Table; Figures 2, 8; Appendix Table 2).

Mammary tissues from serially euthanized animals of both sexes had no or minimal histopathologic changes at all timepoints, including minimal mononuclear or mixed inflammatory infiltrates and mild periglandular edema in 5/17 animals (at 12–16 dpi) for which mammary glandular tissue was evaluated. Mammary skin occasionally showed mild dermal lymphohistiocytic inflammation, epidermal keratinocyte degeneration, and single cell death. Although vRNA was detected by qRT-PCR in mammary gland as early as 4 dpi, staining by IHC or ISH was seen in mammary gland or skin in 8/12 tested female animals (8–16 dpi) and 3/8 tested male animals (12–16 dpi), characterized by multifocal staining with similar distribution as described for the mammary tissues of lethally infected animals (Table; Appendix Table 2).

### Reduced Pathology and No LASV Detection in Surviving Guinea Pigs

**Figure 9:**
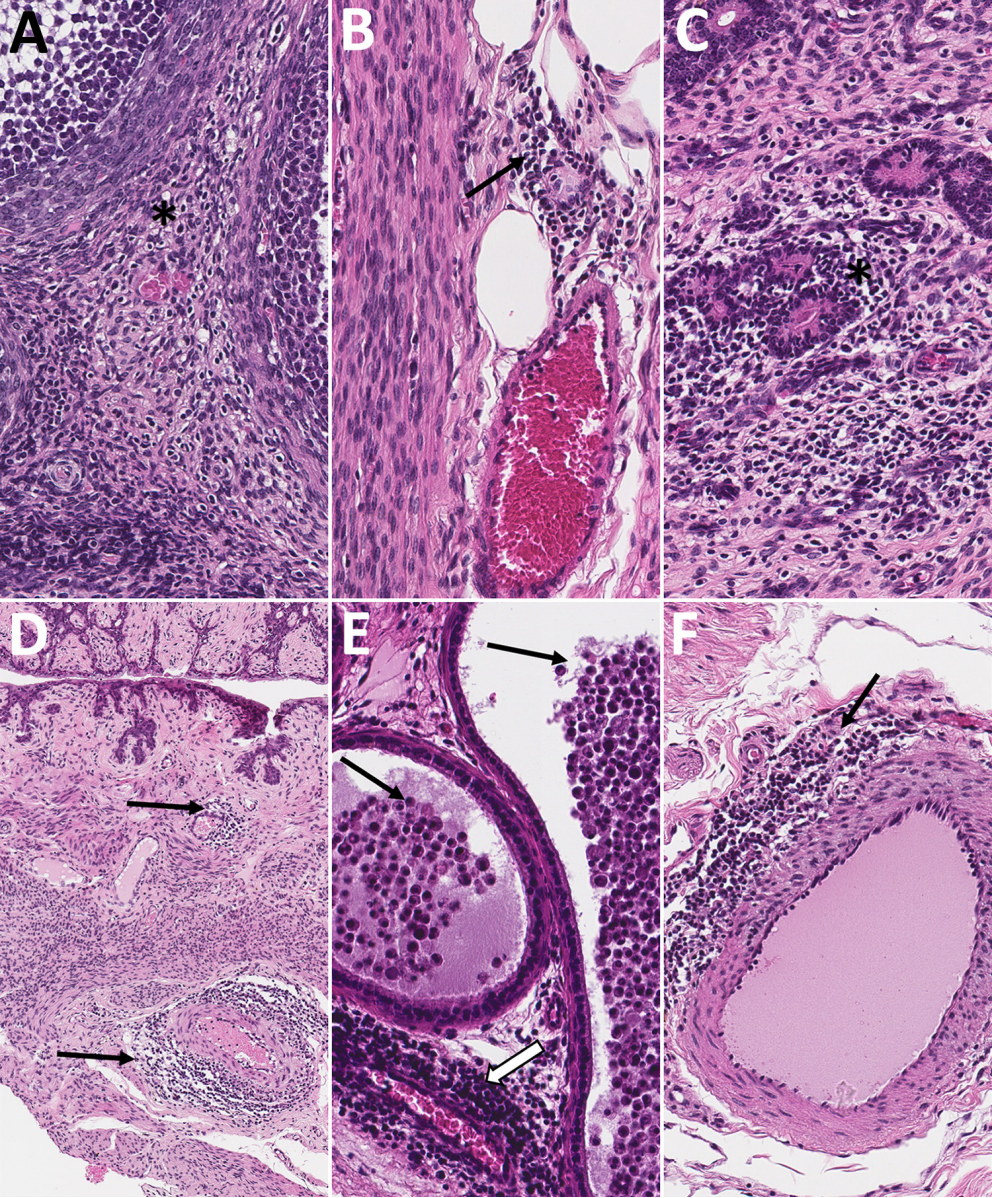
Histopathologic findings in reproductive tissues from strain 13/N guinea pig survivors of Lassa virus strain Josiah infection at 42 days postinfection (dpi) in study of guinea pig model for Lassa virus infection of reproductive tract and considerations for sexual and vertical transmission. Panels A–D depict samples from female guinea pigs; panels E–F depict male samples from male guinea pigs. A) Ovary (42 dpi) with lymphoplasmacytic inflammation in the stroma (asterisk). B) Oviduct (42 dpi) with focal perivascular and interstitial inflammation (arrow). C) Uterus (42 dpi) with endometrial stromal lymphoplasmacytic inflammation (asterisk) around glands. D) Vagina (42 dpi) with lymphocytic inflammation (arrows) around vessels in the vaginal wall. E) Epididymis (42 dpi) with dense perivascular lymphoplasmacytic inflammation around a vessel (white arrow) and heterophils within tubular lumens (black arrows). F) Penile connective tissue (42 dpi) with perivascular lymphocytic inflammation (arrow). Hematoxylin and eosin stain. Original magnification ×40 (panels A–C, E, F), ×10 (panel D).

To assess reproductive tissue pathology and viral persistence in LASV survivors, we histologically examined tissues from 21 LASV-infected guinea pigs (strain Josiah or NJ2015) euthanized at 41–42 dpi. Animals represented 2 outcomes: survival after overt clinical disease or survival without overt clinical disease (Appendix Table 3). Overt disease was defined as weight loss >10%, temperature >39.5°C for >2 consecutive days, or a clinical score of >1 on >1 days. Both untreated and vaccinated surviving animals were included, because animals with and without clinical intervention are clinically relevant for these investigations. Among 12 survivors of overt clinical disease, similar but generally less severe perivascular and interstitial inflammatory changes described for animals in other groups (<28 dpi) were variably present at 41–42 dpi (Table). All animals with inflammation had been infected with LASV-Josiah; those included 4/5 female animals with effects in 2 ovaries, 3 oviducts, 4 uteri, and 4 cervix/vagina and 3 mammary glands and 3/7 male animals with inflammation in 1 tissue each, including 2 epididymides and 1 penis/prepuce. No histopathologic changes were seen in testes or accessory sex glands of surviving male animals (Table; Figures 2, 9; Appendix Table 3). Among 9 surviving animals without overt clinical disease, 1/5 female animals had minimal inflammation in mammary glandular tissue but no inflammation in other reproductive tissues. One of 4 male animals had minimal epididymal inflammation only, and another male survivor had minimal inflammation in the penis/prepuce. Neither viral antigen nor RNA were detected by IHC or ISH in any female or male reproductive tissue tested from surviving animals, regardless of clinical course. We evaluated tissues from 14 survivors (all infected with LASV-Josiah) by qRT-PCR targeting the nucleoprotein; vRNA was not detected in ovaries or testes from 5 survivors without overt signs, whereas low levels of vRNA were detected in 4/4 ovaries (78.4–270 copies/µL) and 4/5 testes (15–50 copies/µL) of 9 survivors after overt disease.

## Discussion

Using guinea pig models of both lethal and nonlethal LF, we characterized reproductive tissue tropism and associated histopathologic changes. In lethal cases, we found widespread reproductive tract infection, but pathology was often subtle or nonspecific, whereas survivors exhibited minimal pathology and no ISH or IHC staining. Furthermore, after infection with a known lethal strain, vRNA was detected in reproductive tract tissues early in infection, preceding the onset of clinical signs or tissue inflammation.

The overall paucity of inflammation in LASV infection could reflect the ability of LASV to replicate in dendritic cells and macrophages and create immune-privileged reservoirs that enable unchecked LASV replication early in infection and subsequent systemic spread, including to the reproductive tract (*28*,*32*,*33*). We detected viral antigen and RNA in multiple reproductive tissues and cell types in male and female animals, including in tissue and cell types previously described for guinea pigs (*32*). ISH, which detects vRNA, was more sensitive than IHC, which detects viral protein; among 36 positive tissues on which both IHC and ISH were performed, results were ISH positive and IHC negative for 18 (50%). That finding indicates that viral RNA might persist or be detectable in tissues even when protein expression is below the threshold of IHC detection. In female animals, vRNA detection was earliest and most abundant in uteri and ovaries; for male animals, vRNA detection was earliest and most extensive in epididymides. Whether LASV has a specific tropism for these tissues or whether their apparent preferential infection is the result of hemodynamic factors during viremia is unclear. At peak viremia, staining was predominantly, but not exclusively, localized in tissues with inflammatory changes; however, staining was also seen in tissues without overt pathology. Mesenchymal cells (interstitial stromal cells and smooth muscle cells) were affected earlier and more commonly than epithelial cells (granulosa cells, endometrial glands, mammary glands, epididymal epithelium and endothelial cells), suggesting that infected fibroblasts could be involved in immune activation and modulation in LASV infection (*34*).

Conversely, in surviving animals, mild inflammation was sometimes present, but vRNA and antigen were not detected by ISH and IHC. However, low-level vRNA was detected by qRT-PCR in about half of survivor gonadal tissues, exclusively in animals that had exhibited overt clinical signs. Those findings suggest substantial viral clearance from reproductive tissues by 42 dpi, although clearance may be incomplete, particularly in more severe cases. Shedding of LASV by humans can be prolonged; even after recovery, virus remains infective in body fluids, including semen, vaginal fluid, urine, and breast milk for long periods (*12*,*19*,*20*). Although we did not directly test genital fluids in this study, IHC or ISH staining was detected in tissues that contribute to genital fluids (testes, epididymides, male accessory sex glands, cervix/vagina) at the latest timepoints examined (23–26 dpi) in lethally infected animals. Altogether, our findings indicate a window of potential risk for sexual transmission of as early as 4 dpi, before the onset of clinical signs, and extending up to 3–4 weeks, or possibly longer, after infection, underscoring the need for continued research on LASV persistence in reproductive tissues and factors influencing the duration of detection.

Our findings have implications for reproductive health, including fertility and pregnancy outcomes, and suggest the potential for both sexual and vertical LASV transmission. We detected LASV in ovarian thecal and granulosa cells, which are the cells that differentiate into the corpus luteum and secrete progesterone that is essential for early pregnancy maintenance in both guinea pigs and humans (*23*). Ovarian infection might lead to early pregnancy loss because of virus-induced progesterone insufficiency and to detrimental effects not only on a current pregnancy but on future fertility (*35*). In uteri, we observed uterine vascular, stromal, and epithelial LASV staining, which has also been previously reported in guinea pigs for LASV and filoviral infections (*32*,*36*). We did not investigate placentas, but others have reported high human placental viral LASV titers and demonstrated viral antigen in trophoblasts (*21*,*22*). Our data indicating LASV predilection for the uterus suggest high risk for direct placental infection, with potential for placental compromise and transplacental transmission to the fetus.

Male reproductive tract infections can also be related to infertility and fetal infection. In our study, viral detection in testes was limited to rare interstitial cells and occurred without associated pathology, possibly because LASV does not principally target the testes or possibly because of the restriction of viral replication by testicular cells with innate antiviral properties (*37*). Epididymides showed more consistent pathology and LASV staining, including epididymal epithelial and interstitial mesenchymal cell staining, as well as apparent staining of leukocytes and intraluminal spermatozoa. Together with occasional staining observed in seminal vesicle and prostate, those findings indicate potential for infection of both spermatozoa and seminal fluid. Although the immune-privileged environment of the testis and epididymis is capable of local defense against microbial pathogens, it also provides a microbial escape from immune surveillance and could prevent or delay clearance; potentially, adverse effects on male fertility, as well as sexual transmission, even after long periods postinfection, could be possible (*38*,*39*).

Breastfeeding poses another potential risk for LASV transmission to infants. Shedding in milk and high viral titers with viral immunostaining in mammary glandular tissue has been demonstrated in humans (*21*,*22*). In addition, natural and experimental infections through skin exposure have been reported (*13*–*17*,*40*). We found viral antigen and RNA localized to both mammary glandular epithelium and overlying epidermis, indicating that both milk and skin contact might be possible sources of infant infection during breastfeeding. However, no specific study links breastfeeding to LASV transmission, and our findings only highlight the need for additional interdisciplinary research and surveillance efforts to understand these risks.

In conclusion, our findings in a guinea pig model demonstrate that LASV infects both female and male reproductive tracts as early as 4 dpi, indicating a potential risk for early sexual and vertical transmission, including through breastfeeding, even during the incubation period before clinical signs are apparent. This model further suggests that transmission risk could persist for at least 4 weeks and possibly longer, warranting additional studies of human cases to better inform evidence-based guidance for survivors regarding abstinence or protected-sex practices as part of behavioral interventions to reduce transmission, considerations that are particularly relevant given the current lack of available LASV-specific vaccines or therapeutics. More broadly, this study highlights critical gaps in our understanding of long-term sequelae and tissue reservoirs for LASV and other viral hemorrhagic fevers in survivors, as well as the need for focused investigation into infection during pregnancy and its effects on maternal, placental, and fetal health. Such knowledge is essential for refining survivor counseling, infection prevention strategies, and public health preparedness in LASV-endemic settings.

Appendix Additional information about guinea pig model for Lassa virus infection of reproductive tract and considerations for sexual and vertical transmission
