# Supplementary material for: Guinea Pig Model for Lassa Virus Infection of Reproductive Tract and Considerations for Sexual and Vertical Transmission
Source: Emerg Infect Dis. 2025 Dec;31(12):2265–78. doi: 10.3201/eid3112.250396 (PMC12782183; doi:10.3201/eid3112.250396)
Supplement: Appendix — Additional information about guinea pig model for Lassa virus infection of reproductive tract and considerations for sexual and vertical transmission [file 25-0396-Techapp-s1.pdf]

Article DOI: https://doi.org/10.3201/eid3112.250396

EID cannot ensure accessibility for supplementary materials supplied by authors. Readers who have difficulty accessing supplementary content should contact the authors for assistance.

Guinea Pig Model for Lassa Virus Infection of Reproductive Tract and Considerations for Sexual and Vertical Transmission

Appendix

Supplementary Methods

RT-qPCR

RNA was extracted from homogenized tissue samples (ovary, uterus, testis, epididymis, mammary gland) and urogenital swabs (intravaginal or preputial) [swab data previously reported in (*1*)] using the MagMAX-96 Total RNA Isolation Kit (Thermo Fisher Scientific) on a 96-well ABI MagMAX extraction platform with a DNase-I treatment step, according to the manufacturer's instructions. RNA was quantified by a reverse transcription-quantitative PCR (RT-qPCR) targeting a strain-specific nucleoprotein gene sequence (primer and probe sequences available on request from the authors) and normalized to 18S RNA levels. We determined viral small (S) segment copy numbers using standards prepared from in vitro–transcribed S segment RNA.

Histopathology, Immunohistochemistry (IHC), and In situ hybridization (ISH)

Representative sections of female (ovary, oviduct, uterus and cervix/ vagina) and male (testis, epididymis, seminal vesicle/ prostate and penis/prepuce) reproductive tissues, and mammary gland, as available, were evaluated by histopathology. Tissue specimens were fixed in 10% neutral buffered formalin and gamma-irradiated (5×10^6 rad) and processed for routine paraffin histology. Sections were cut at 4 µm, mounted on glass slides, and stained with hematoxylin-eosin for histopathologic evaluation, or by immunohistochemistry (IHC) or in situ hybridization (ISH) for LASV antigens or RNA, respectively. IHC and ISH were performed in a

subset of tissues chosen based on representation of timepoints, presence of tissue pathology, and/or viral detection by RT-qPCR. Two veterinary pathologists visually assessed pathologic changes in tissues, and staining by LASV IHC and ISH. Pathologic changes were described qualitatively and scored, and viral antigen and RNA detection were scored semiquantitatively as follows: absent (0), minimal (1), mild (2), moderate (3), severe (4).

IHC assay for LASV used a mouse monoclonal antibody mix (SPR628) targeting LASV nucleoprotein and glycoprotein at 1:1,000 (CDC), as previously described (*2*,*3*). Colorimetric detection of attached antibodies was performed using the Mach 4 AP polymer kit (Biocare Medical) at room temperature, except for heat-induced epitope retrieval. Appropriate negative control serum was run in parallel. Slides were counterstained with Mayer's hematoxylin (Poly Scientific) and blued in lithium carbonate (Poly Scientific). Positive controls included formalin-fixed, paraffin-embedded (FFPE) Vero-E6 cells infected with LASV.

To localize LASV RNA in FFPE tissues, an ISH assay using an RNAscope probe (Advanced Cell Diagnostics) targeting the polymerase gene of LASV was performed, as previously described (*4*,*5*). All tissues were tested in parallel with appropriate controls. An FFPE cell culture block, infected with LASV, with application of probe served as the positive control, while the same block without probe application served as the negative control. Specificity testing for LASV probe was verified by testing of FFPE tissues from clinically or genetically similar viruses.

References

1. Welch SR, Genzer SC, Coleman-McCray JD, Harmon JR, Scholte FEM, Montgomery JM, et al. Viral RNA and infectious virus in mucosal specimens from guinea pigs modelling early phases of lethal and non-lethal Lassa fever. Emerg Microbes Infect. 2022;11:1390–3. PubMed https://doi.org/10.1080/22221751.2022.2071637

2. Gary JM, Welch SR, Ritter JM, Coleman-McCray J, Huynh T, Kainulainen MH, et al. Lassa Virus Targeting of Anterior Uvea and Endothelium of Cornea and Conjunctiva in Eye of Guinea Pig Model. Emerg Infect Dis. 2019;25:865–74. PubMed https://doi.org/10.3201/eid2505.181254

3. Huynh T, Gary JM, Welch SR, Coleman-McCray J, Harmon JR, Kainulainen MH, et al. Lassa virus antigen distribution and inflammation in the ear of infected strain 13/N Guinea pigs. Antiviral Res. 2020;183:104928. PubMed https://doi.org/10.1016/j.antiviral.2020.104928

4. Wang F, Flanagan J, Su N, Wang L-C, Bui S, Nielson A, et al. RNAscope: a novel in situ RNA analysis platform for formalin-fixed, paraffin-embedded tissues. J Mol Diagn. 2012;14:22–9. PubMed https://doi.org/10.1016/j.jmoldx.2011.08.002

5. Bhatnagar J, Gary J, Reagan-Steiner S, Estetter LB, Tong S, Tao Y, et al. Evidence of Severe Acute Respiratory Syndrome Coronavirus 2 Replication and Tropism in the Lungs, Airways, and Vascular Endothelium of Patients With Fatal Coronavirus Disease 2019: An Autopsy Case Series. J Infect Dis. 2021;223:752–64. PubMed https://doi.org/10.1093/infdis/jiab039

Appendix Table 1. Lethal endpoint: signalment, vaccine status, clinical progression, pathologic changes and viral detection in reproductive tract tissues of strain 13/N guinea pigs succumbing to lethal LASV strain Josiah*

Animal ID	Age at D0 (days)	Sex	DPI	Vaccine type	Max weight loss	Max Temp (°C)	Max score	Days >0 score	Ovary or testis vRNA (copies/µL)	H&E / PCR / IHC / ISH					
										Ovary or testis	Oviduct or Epididymis	Uterus or Sem. Vesicle / Prostate	Cervix/ Vagina or Penis/ Prepuce	Mammary gland	Mammary skin
2833–1-7	1394	F	14	NA	−23.4%	39.9	ND	ND	ND	2/○/2/○	2/○/1/○	2/○/3/○	1/○/1/○	○/○/○/○	○/○/○/○
2833–1-10	840	F	17	NA	−19.3%	40.4	ND	ND	ND	1/○/1/○	1/○/1/○	1/○/2/○	−/○/−/○	○/○/○/○	○/○/○/○
2833–1-9	872	F	20	NA	−21.5%	39.8	ND	ND	ND	1/○/1/○	2/○/2/○	1/○/2/○	−/○/−/○	○/○/○/○	○/○/○/○
2833–5-17	483	F	23	NA	−24.8%	40.0	12	5	2.2×10^{7}	2/+/2/3	2/○/2/3	3/○/3/3	1/○/2/3	2/○/2/2	−/○/2/2
2833–5-11	751	F	25	ɣ-VRP	−23.2%	39.5	12	8	1.1×10^{6}	1/+/2/○	2/○/2/○	2/○/3/○	1/○/1/○	1/○/2/○	−/○/−/○
2833–5-16	900	F	28	NA	−27.7%	39.7	12	11	3.4×10^{4}	−/+/−/−	−/○/−/−	1/○/−/1	−/○/−/−	−/○/−/○	−/○/−/○
2833–1-11	165	M	17	NA	−18.5%	39.5	ND	ND	ND	−/○/−/−	−/○/−/−	−/○/−/1	○/○/○/○	○/○/○/○	○/○/○/○
2833–1-12	165	M	18	NA	−18.8%	40.5	ND	ND	ND	−/○/−/−	−/○/1/2	−/○/−/1	○/○/○/○	○/○/○/○	○/○/○/○
2833–5-13	794	M	23	ɣ-VRP	−21.0%	39.3	12	7	3.4×10^{3}	−/+/−/1	3/○/2/2	1/○/1/○	1/○/−/○	○/○/○/○	○/○/○/○
2833–5-20	591	M	23	NA	−22.8%	39.6	12	7	4.3×10^{3}	−/+/−/−	4/○/2/2	−/○/−/○	−/○/−/○	1/○/−/○	1/○/−/○
2833–5-19	782	M	26	NA	−24.8%	39.3	12	8	2.5×10^{3}	−/+−/1	4/○/2/2	−/○/−/○	−/○/−/○	○/○/○/○	○/○/○/○

*Animals lethally infected with LASV-Josiah strain (subcutaneously, target dose of 10^{4} FFU) and euthanized due to severe clinical disease between 14–28 dpi. LASV vRNA loads in gonadal tissue (testis or ovary) were measured by RT-qPCR (N copy no./µL RNA). Results: +, positive; −, negative/no lesion; ○, indicates that the tissue was not tested. H&E: extent of inflammatory changes seen in hematoxylin-eosin stained tissue sections. IHC: extent of LASV antigen detection by immunohistochemistry. ISH: extent of LASV RNA detection by in situ hybridization. Scoring for H&E, IHC, ISH: 1, minimal; 2, mild; 3, moderate; 4, severe. D0, day of challenge; DPI, days post-infection; ND, not determined; VRP, viral replicon particle vaccine; ɣ-VRP, replication incompetent VRP (treated with 5×10^{6} rads of gamma-irradiation) 28 d pre-challenge.

Appendix Table 2. Early infection: signalment, clinical progression, pathologic changes and viral detection in reproductive tract tissues of strain 13/N guinea pigs serially euthanized up to 16 d after LASV strain Josiah infection.

Animal ID	Age at D0 (d)	Sex	DPI	Max Weight loss	Max Temp (°C)	Max score	Days >0 score	Ovary or testis vRNA (copies/µL)	Uterus or Epididymis vRNA (copies/µL)	H&E / PCR / IHC / ISH					
										Ovary or Testis	Oviduct or Epididymis	Uterus or Sem. Vesicle / Prostate	Cervix/ Vagina or Penis/ Prepuce	Mammary gland	Mammary skin
3073–1-26	428	F	2	−1.1%	37.4	0	0	BLD	BLD	−/−/−/○	−/○/−/○	−/−/−/○	○/○/○/○	○/−/○/○	○/○/○/○
3073–1-27	578	F	2	−3.8%	37.6	0	0	BLD	BLD	−/−/−/−	−/○/−/−	−/−/−/−	○/○/○/○	○/−/○/○	○/○/○/○
3073–1-28	798	F	2	−0.3%	37.2	0	0	BLD	BLD	−/−/−/○	−/○/−/○	−/−/−/○	−/○−/○	−/−/−/○	−/○/−/○
3073–1-31	454	F	4	−0.4%	37.5	0	0	BLD	BLD	−/−/−/○	−/○/−/○	−/−/−/○	−/○/−/○	−/−/−/○	−/○/−/○
3073–1-32	567	F	4	−0.2%	36.8	0	0	BLD	BLD	−/−/−/−	−/○/−/−	−/−/−/1	−/○/−/1	○/−/○/○	○/○/○/○
3073–1-33	985	F	4	−4.6%	37.5	0	0	BLD	BLD	−/−/−/−	−/○/−/−	−/−/−/1	−/○/−/1	−/+/−/○	−/○/−/○
3073–1-36	383	F	8	−2.7%	38.6	0	0	3.4×10^4	6.9×10^3	−/+/−/−	−/○/−/−	−/+/−/1	−/○/−/1	−/+/−/○	−/○/−/○
3073–1-37	613	F	8	−5.4%	37.5	0	0	8.5×10^4	2.2×10^3	−/+/1/○	−/○/−/○	−/+/−/○	−/○/−/○	−/−/1/1	−/○/1/1
3073–1-38	811	F	8	−3.0%	38.0	0	0	7.3×10^5	5.5×10^4	−/+/1/○	−/○/−/○	−/+/1/○	−/○/−/○	−/+/1/1	−/○/1/1
3073–1-41	365	F	12	−7.7%	40.1	0	0	2.9×10^5	1.1×10^6	−/+/3/3	−/○/1/3	1/+/3/3	−/○/3/○	1/+/1/○	1/○/2/○
3073–1-42	577	F	12	−3.6%	39.2	0	0	7.6×10^6	9.6×10^6	−/+/2/○	−/○/−/○	1/+/3/○	−/○/1/○	1/+/1/○	1/○/2/○
3073–1-43	845	F	12	−2.9%	39.5	0	0	3.7×10^6	6.4×10^6	−/+/2/○	1/○/1/○	1/+/3/○	−/○/1/○	1/+/2/○	1/○/2/○
3073–1-46	394	F	16	−9.8%	41.2	0	0	1.1×10^6	7.3×10^3	−/+/1/○	−/○/−/○	2/+/1/○	2/○/2/○	−/+/2/○	1/○/1/○
3073–1-47	577	F	16	−7.2%	39.5	1	1	1.8×10^4	5.6×10^5	−/+/−/○	−/○/−/○	2/+/2/○	1/○/2/○	−/+/−/○	1/○/1/○
3073–1-48	831	F	16	−4.6%	39.3	0	0	6.3×10^3	1.8×10^3	2/+/2/○	−/○/1/○	2/+/2/○	○/○/○/○	1/+/1/○	1/○/2/○
3073–1-29	385	M	2	−0.4%	37.8	0	0	BLD	BLD	−/−/−/−	−/−/−/−	−/○/−/−	○/○/○/○	−/−/−/○	−/○/−/○
3073–1-30	654	M	2	0.3%	38.1	0	0	BLD	BLD	−/−/−/○	−/−/−/○	−/○/−/○	○/○/○/○	−/−/−/○	−/○/−/○
3073–1-34	383	M	4	−0.2%	37.8	0	0	BLD	BLD	−/−/−/○	−/−/−/○	−/○/−/○	○/○/○/○	−/−/−/○	−/○/−/○
3073–1-35	558	M	4	−1.8%	37.8	0	0	BLD	BLD	−/−/−/○	−/−/−/○	−/○/−/○	○/○/○/○	−/−/−/○	−/○/−/○
3073–1-39	400	M	8	0.2%	37.6	0	0	1.0×10^5	3.4×10^4	−/+/−/−	−/+/−/1	−/○/−/−	○/○/○/○	○/+/○/○	○/○/○/○
3073–1-40	646	M	8	−4.5%	37.9	0	0	1.1×10^2	7.4×10^2	−/+/−/−	−/+/−/−	−/○/−/−	○/○/○/○	○/+/○/○	○/○/○/○
3073–1-44	405	M	12	−5.6%	39.2	0	0	2.6×10^4	3.3×10^3	−/+/−/1	−/+/−/1	−/○/−/1	○/○/○/○	1/+/2/○	1/○/2/○
3073–1-45	594	M	12	−4.9%	39.2	0	0	9.8×10^3	1.0×10^3	−/+/−/1	−/+/−/−	−/○/−/−	○/○/○/○	○/+/○/○	−/○/1/○
3073–1-49	400	M	16	−11.0%	39.9	2	2	5.6×10^3	5.0×10^4	−/+/−/−	1/+/2/2	−/○/−/1	○/○/○/○	○/+/○/○	1/○/1/○
3073–1-50	663	M	16	−7.6%	40.1	5	3	9.9×10^2	5.6×10^1	−/+/−/−	−/+/−/−	−/○/−/1	○/○/○/○	○/+/○/○	−/○/−/○

*Animals inoculated subcutaneously with LASV-Josiah strain (target dose: 10^4 FFU) and serially euthanized between 2–16 dpi. vRNA loads in tissue measured by RT-qPCR (N copy no./µL RNA). Results: +, positive; −, negative/no lesion; ○, indicates that the tissue was not tested. H&E: extent of inflammatory changes seen in hematoxylin-eosin stained tissue sections. IHC: extent of LASV antigen detection by immunohistochemistry. ISH: extent of LASV RNA detection by in situ hybridization. Scoring for H&E, IHC, ISH: 1, minimal; 2, mild; 3, moderate; 4, severe. BLD, below the limit of detection; D0, day of challenge; DPI, days post-infection.

Appendix Table 3. Survivors: signalment, vaccine status, clinical progression, pathologic changes and viral detection in reproductive tract tissues of strain 13/N guinea pigs that survived LASV (strain Josiah or NJ2015) infection.

Animal ID	Age at D0 (d)	Sex	Vaccine type	LASV strain	DPI	Overt* disease	Max Weight loss	Max Temp (°C)	Max score	Days >0 score	Ovary or testis vRNA (copies/μL)	H&E / PCR / IHC / ISH					
												Ovary or testis	Oviduct or Epididymis	Uterus or Sem. Vesicle / Prostate	Cervix/ Vagina or Penis/ Prepuce	Mammary gland	Mammary skin
2833–1-1	1433	F	NA	NJ	41	No	−9.7%	38.5	ND	ND	ND	−/○/−/−	−/○/−/−	−/○/−/−	−/○/−/−	○/○/○/○	○/○/○/○
2833–1-3	872	F	NA	NJ	41	No	−4.0%	39.3	ND	ND	ND	−/○/−/○	−/○/−/○	−/○/−/○	−/○/−/○	○/○/○/○	○/○/○/○
2833–5-1	794	F	VRP	Josiah	42	No	−2.9%	38.1	0	0	BLD	−/−/−/○	−/○/−/○	−/○/−/○	−/○/−/○	−/○/−/○	−/○/−/○
2833–5-2	751	F	VRP	Josiah	42	No	−0.4%	39.0	0	0	BLD	−/−/−/○	−/○/−/○	−/○/−/○	−/○/−/○	−/○/−/○	−/○/−/○
2833–5-3	402	F	VRP	Josiah	42	No	−4.6%	38.1	0	0	BLD	−/−/−/○	−/○/−/○	−/○/−/○	−/○/−/○	1/○/−/−	−/○/−/−
2833–1-4	840	F	NA	NJ	41	Yes	−12.6%	38.8	ND	ND	ND	−/○/−/○	−/○/−/○	−/○/−/○	−/○/−/○	○/○/○/○	○/○/○/○
2833–5-6	900	F	VRP-PE	Josiah	42	Yes	−14.8%	39.3	0	0	7.8×10^{1}	−/+/−/−	1/○/−/−	1/○/−/−	1/○/−/−	1/○/−/−	−/○/−/−
2833–5-7	794	F	VRP-PE	Josiah	42	Yes	−16.5%	39.7	2	6	2.2×10^{3}	−/+/−/−	1/○/−/−	2/○/−/−	2/○/−/−	1/○/−/−	−/○/−/−
2833–5-12	325	F	ɣ-VRP	Josiah	42	Yes	−14.3%	39.7	3	5	9.7×10^{2}	1/+/−/−	1/○/−/−	1/○/−/−	1/○/−/−	1/○/−/−	−/○/−/−
2833–5-18	325	F	NA	Josiah	42	Yes	−11.7%	39.2	1	1	2.7×10^{3}	1/+/−/−	−/○/−/−	1/○/−/−	2/○/−/−	−/○/−/○	−/○/−/○
2833–1-2	1341	M	NA	NJ	42	No	−2.8%	39.6	ND	ND	ND	−/○/−/○	−/○/−/○	−/○/−/○	○/○/○/○	○/○/○/○	○/○/○/○
2833–1-8	840	M	NA	Josiah	41	No	−1.0%	38.8	ND	ND	ND	−/○/−/−	1/○/−/−	−/○/−/−	○/○/○/○	○/○/○/○	○/○/○/○
2833–5-4	751	M	VRP	Josiah	42	No	−4.7%	38.4	0	0	BLD	−/−/−/○	−/○/−/○	−/○/−/−	1/○/−/−	○/○/○/○	○/○/○/○
2833–5-5	325	M	VRP	Josiah	42	No	−0.1%	38.2	0	0	BLD	−/−/−/○	−/○/−/○	−/○/−/○	−/○/−/○	○/○/○/○	−/○/−/−
2833–1-5	165	M	NA	NJ	42	Yes	−7.2%	39.9	ND	ND	ND	−/○/−/○	−/○/−/○	−/○/−/○	○/○/○/○	○/○/○/○	○/○/○/○
2833–1-6	165	M	NA	NJ	42	Yes	−16.4%	39.9	ND	ND	ND	−/○/−/○	−/○/−/○	−/○/−/○	○/○/○/○	○/○/○/○	○/○/○/○
2833–5-8	483	M	VRP-PE	Josiah	42	Yes	−18.6%	39.1	3	12	5.0×10^{2}	−/+/−/−	−/○/−/−	−/○/−/○	−/○/−/○	−/○/−/○	−/○/−/○
2833–5-9	322	M	VRP-PE	Josiah	42	Yes	−23.6%	39.7	7	22	5.5×10^{1}	−/+/−/−	−/○/−/−	−/○/−/−	1/○/−/−	−/○/−/○	−/○/−/○
2833–5-10	320	M	VRP-PE	Josiah	42	Yes	−21.5%	39.5	8	18	1.5×10^{1}	−/+/−/−	−/○/−/−	−/○/−/○	−/○/−/○	−/○/−/○	−/○/−/○
2833–5-14	746	M	ɣ-VRP	Josiah	42	Yes	−21.4%	39.1	9	25	4.1×10^{2}	−/+/−/−	2/○/−/−	−/○/−/○	−/○/−/○	−/○/−/○	−/○/−/○
2833–5-15	402	M	ɣ-VRP	Josiah	42	Yes	−21.5%	39.2	7	20	BLD	−/−/−/−	2/○/−/−	−/○/−/−	−/○/−/−	−/○/−/○	−/○/−/○

Animals inoculated subcutaneously with LASV (target dose: 10^{4} FFU) and euthanized at study end (41–42 dpi). *Inclusion criteria for animals with overt signs include: weight loss ≥10%, temperature ≥39.5°C for two or more consecutive days and/or presence of clinical signs (i.e., clinical score ≥1 on one or more days). vRNA loads in gonadal tissue measured by RT-qPCR (N copy no./μL RNA). Results: +, positive; −, negative/no lesion; ○, indicates that the tissue was not tested. H&E: extent of inflammatory changes seen in hematoxylin-eosin stained tissue sections. IHC: extent of LASV antigen detection by immunohistochemistry. ISH: extent of LASV RNA detection by in situ hybridization. Scoring for H&E, IHC, ISH: 1, minimal; 2, mild; 3, moderate; 4, severe. BLD, below the limit of detection; D0, day of challenge; DPI, days post-infection; ND, not determined; NA, not applicable (i.e., not vaccinated); NJ, strain NJ2015; VRP (viral replicon particle vaccine), VRP given 28 d pre-challenge; VRP-PE, VRP administered 1 d post-exposure; ɣ-VRP, replication incompetent VRP (treated with 5×10^{6} rads of gamma-irradiation) given 28 d pre-challenge.
